# Novel histone deacetylase inhibitor N25 exerts anti-tumor effects and induces autophagy in human glioma cells by inhibiting HDAC3

**DOI:** 10.18632/oncotarget.20744

**Published:** 2017-09-08

**Authors:** Xin-Yuan Sun, Yue Qu, An-Ran Ni, Gui-Xiang Wang, Wei-Bin Huang, Zhong-Ping Chen, Zhu-Fen Lv, Song Zhang, Holly Lindsay, Sibo Zhao, Xiao-Nan Li, Bing-Hong Feng

**Affiliations:** ^1^ Department of Pharmacology, College of Pharmacy, Guangdong Pharmaceutical University, Guangzhou, China; ^2^ Department of Neurosurgery/Neuro-oncology, Sun Yat-sen University Cancer Center, Guangzhou, China; ^3^ Guangdong Provincial Key Laboratory of Advanced Drug Delivery, Guangdong Pharmaceutical University, Guangzhou, China; ^4^ Department of Clinical Pharmacy, Puning People's Hospital, Puning, China; ^5^ Department of Pharmacy, The First People’s Hospital of Guangyuan, Guangyuan, China; ^6^ Preclinical Neuro-Oncology Research Program, Texas Children’s Cancer Center, Department of Pediatrics, Baylor College of Medicine, Houston, TX, USA

**Keywords:** histone deacetylase inhibitor, N25, autophagy, glioma, HDAC3

## Abstract

N25, a novel histone deacetylase inhibitor, was created through structural modification of suberoylanilide hydroxamic acid. To evaluate the anti-tumor activity of N25 and clarify its molecular mechanism of inducing autophagy in glioma cells, we investigated its *in vitro* anti-proliferative effect and *in vivo* anticancer effect. Moreover, we detected whether N25 induces autophagy in glioma cells by transmission electron microscope and analyzed the protein expression level of HDAC3, Tip60, LC3 in glioma samples by western blot. We additionally analyzed the protein expression level of HDAC3, Tip60, ULK1 (Atg1), and Beclin-1 (Atg6) after treatment with N25 in glioma cells. Our results showed that the anti-tumor activity of N25 in glioma cells is slightly stronger than SAHA both i*n vitro* and *in vivo*. We found that N25 induced autophagy, and HDAC3 was significantly elevated and Tip60 and LC3 significantly decreased in glioma samples compared with normal brain tissues. Nevertheless, N25 inhibited HDAC3 and up-regulated the protein expression of Tip60, ULK1 (Atg1), and Beclin-1 (Atg6) after treatment of glioma cells with N25. In conclusion, these data suggest that N25 has striking anti-tumor activity in part due to inhibition of HDAC3. Additionally, N25 may induce autophagy through inhibiting HDAC3.

## INTRODUCTION

Glioma is both the most common and lethal primary brain tumor which occurs in adults and children [1, 2]. The median survival rate is only 13-16 months after standard therapy and more than 70% of glioblastoma patients die within two years of diagnosis [3, 4]. Therefore, development of novel therapeutic drugs and new treatment strategies is essential for improving glioblastoma survival.

Epigenetic changes play a key role in the occurrence and development of cancer [5–7] and are a hot spot in international cancer research. Histone acetylation and deacetylation are important ways of epigenetic modifications [8]. Histone deacetylases function together with histone acetyltransferases to accurately control gene expression by altering nucleosome conformation, and affecting the stability of several large transcription factor complexes [9]. Numerous studies have confirmed that various human cancers are associated with HDACs overexpression and that histone acetylation imbalance caused by enhanced intra-tumoral HDACs activity affects the occurrence and development of cancer [10–12]. Therefore, HDACs are a promising target for cancer therapy.

Histone deacetylases comprise a family of 18 proteins in humans, consisting of class I proteins (HDAC1, 2, 3 and 8), class IIa proteins (HDAC4, 5, 7, and 9), class IIb proteins (HDAC6 and 10), class III proteins (sirtuins 1-7) and class IV proteins (HDAC11). These enzymes remove acetyl groups from lysine on histones and other proteins [13].

Histone deacetylase inhibitors (HDACi) represent a class of anticancer drugs. Suberoylanilide hydroxamic acid (SAHA), also known as vorinostat, is a synthetic hydroxamic acid that inhibits class I and II HDACs through binding of its hydroxamic acid group with a zinc atom at the bottom of the catalytic cavity, and causing histone acetylations within transcription factors [14, 15]. SAHA is the first drug of this type that has been approved by the US Food and Drug Administration for the treatment of cutaneous T-cell lymphoma [16]. N25 (N1-(2, 5-dimethoxyphenyl)-N8- hydroxyoctanediamide) was obtained through structural modification of SAHA and has been patented for several years. Previous studies demonstrated that N25 is a novel histone deacetylase inhibitor with stronger pharmacological activity than SAHA in several cancer cells [17].

HDAC3, a member of the HDACs family, has been reported to be expressed in almost all human tissues including brain, and usually is up-regulated in solid tumors [18]. A meta-analysis of a variety of human cancers revealed that HDAC3 is one of the most frequently up-regulated genes in cancer cells [19]. Additionally, altered HDACs expressions is implicated in the pathogenesis of glioma [20].

It is generally known that induction of caspase-dependent apoptosis (type I cell death) is a major mechanism by which most chemotherapeutic drugs and radiation kill tumor cells. In recent years, induction of autophagic cell death (type II cell death), one type of nonapoptotic programmed cell death, has been extensively studied [21].

Autophagy is a process of self-degradation of cellular components [22]. Autophagy is characterized by a cell without nuclear condensation during the process of cellular self-digestion in which cellular constituents are engulfed in specialized double-membrane structures called autophagosomes. Autophagosomes then fuse with lysosomes to form autolysosomes for degradation of their cargos and regeneration of nutrients [21, 23]. Microtubule-associated protein 1 light chain 3 (LC3) is required for the elongation of autophagosomes. LC3 has two forms: LC3-I and LC3-II. LC3-II is the most reliable marker for quantification of the level of autophagy in cells, which increases due to the conversion of LC3-I to LC3-II during autophagy.

Autophagy has been proposed to have a dual role, either killing or protecting the cells, depending on the stage of the disease or the surrounding cellular environment [24]. Therefore, assessing the role of autophagy in a context-dependent manner is crucial, especially when considering if autophagy-targeting can be used as anticancer therapy.

ULK1 and Beclin-1 are homologous proteins of Atg1 and Atg6 in mammals, respectively. ULK1, which is required for autophagy, is directly acetylated and stimulated after activation of acetyltransferase Tip60 [25]. Additionally, ULK1 induces autophagy by phosphorylating Beclin-1 and activating VPS34 lipid kinase [26]. Another study showed that knockdown of HDAC3 in cells could increase the level of acetylation of Tip60 [27]. Therefore, we proposed a hypothesis that N25 induces autophagy probably through a new mechanism of upregulating Tip60 by inhibiting HDAC3, causing upregulation of ULK1 (Atg1) and Beclin-1 (Atg6), and resulting in induction of glioma cell autophagy.

In this research, we studied the antitumor activity of N25 *in vitro* and *in vivo*. We also validated whether N25 induces autophagy and detected the changes of crucial proteins in glioma samples. More importantly, we conducted preliminary investigation on the molecular mechanism of N25 induced autophagy. Our study demonstrated that N25 has striking anti-tumor activity and was the first to show that HDACi induces autophagy through inhibiting HDAC3.

## RESULTS

### N25, a novel HDACi, inhibits the cell viability in glioma cells

**Figure 1:**
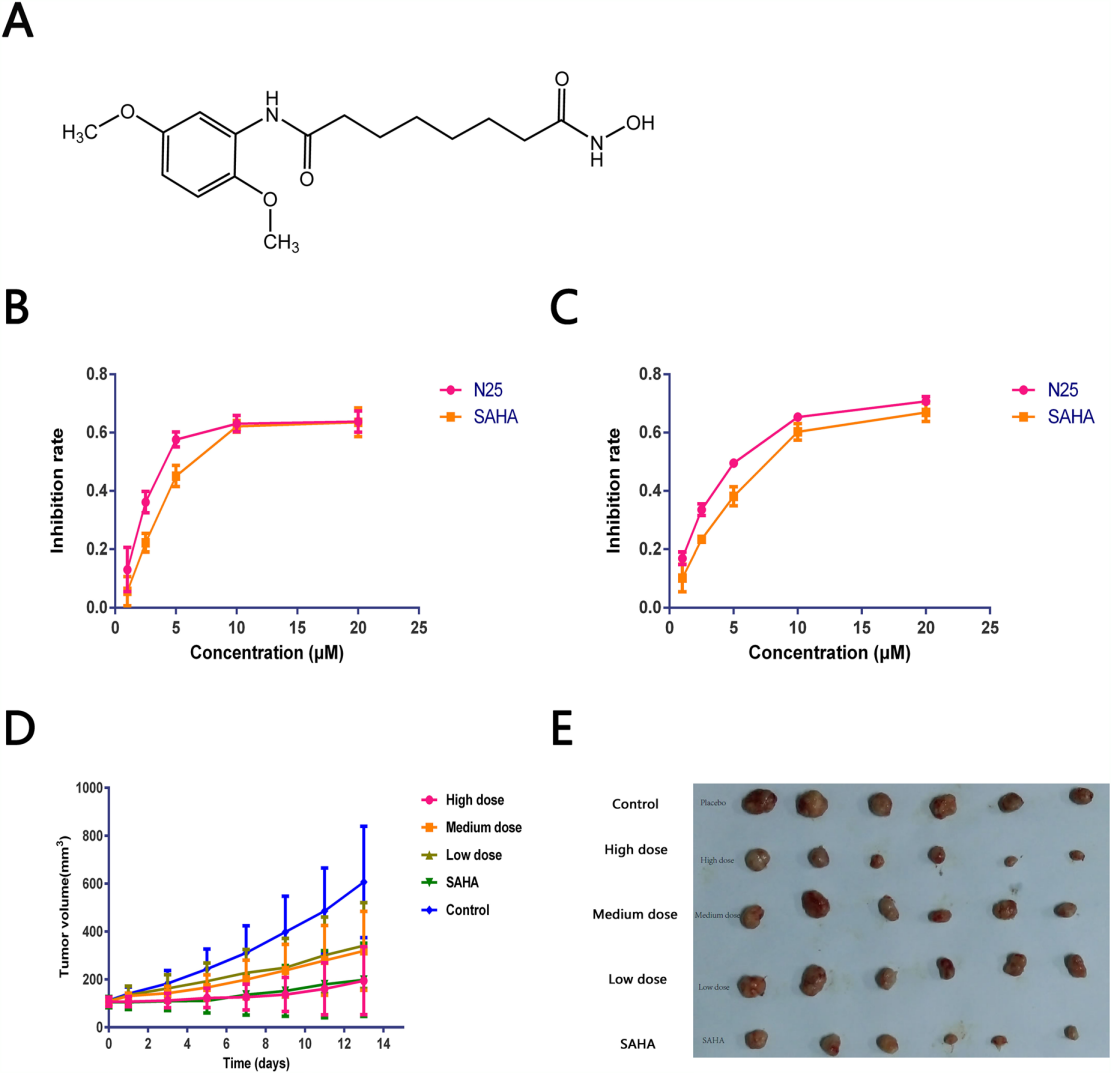
Anti-tumor effects of N25 **(A)** The chemical structure of N25. **(B** and **C)** U87-MG or U251 was exposed to gradually increasing concentrations of N25 or SAHA for 48 hours. Cell viability was detected by using the Cell Counting Kit-8. Cell growth was significantly inhibited after treatment with N25 in U87-MG and U251 cells (P<0.05). **(D)** U87-MG was subcutaneously injected into nude mice. Mice were treated with one of three doses of N25, SAHA, or 0.9% NaCl. The tumor volume was measured (P<0.05). **(E)** Xenograft tumor picture.

To explore the anti-proliferative activity of N25 on glioma cells, U87-MG and U251 cells were chosen for analyses. Cells were exposed to gradually increasing concentrations of N25 for 48 hours. Subsequently, cell viabilities were detected with Cell Counting Kit-8. The growth-inhibitory curves of U87-MG and U251 were plotted (Figure 1B, 1C), and IC_50_ values were calculated. The IC50 of N25 and SAHA were 2.55 μM and 3.58 μM respectively in U87-MG. The IC50 of N25 and SAHA were 5.63 μM and 8.13 μM respectively in U251. The growth-inhibitory curves demonstrated that N25 suppressed U87-MG and U251 viability more significantly than SAHA at relatively low concentration *in vitro*.

### N25 suppresses tumor growth in a glioma xenograft tumor model

To evaluate the anticancer effect of N25 *in vivo*, an anti-tumor study was performed using a nude mice xenograft model. 3-5×10^6^ U87-MG cells were subcutaneously implanted into the right armpit of nude mice. Two weeks after implantation, mice bearing similar sizes of tumors were divided into five groups and there are 6 nude mice in each group. N25 at one of three doses (24, 48, or 96 mg/kg), SAHA (96 mg/kg) or Control (0.9% NaCl). After intragastric drug administration over two weeks, the nude mice were sacrificed and the xenografted tumors from treatment and control groups harvested, dissected and weighed. The average tumor volume of each treatment group was significantly lower than that of the control group on day 14. Tumor growth curve and therapeutic effects are shown (Figure 1D, Table 1). Significant tumor growth inhibition was observed in the N25 treatment group compared to the control group, with tumor inhibition rate of 68% in the 96 mg/kg N25 group.

**Table 1:** The therapeutic effect of N25 in a U87 xenograft tumor model (n=6)

Group	Dose	Average weight		Tumor weight (x±SD)	TIR (%)
		d0	d14		
Control	0.9% Nacl	20.1	19.5	0.93±0.36	0
High dose	96 mg/kg	20.2	18.8	0.30±0.24	68%±0.26^**^
Medium	48 mg/kg	20.1	19.2	0.52±0.26	45%±0.21^*^
Low dose	24 mg/kg	19.8	19.1	0.60±0.30	36%±0.16^*^
SAHA	96 mg/kg	19.9	19.2	0.32±0.21	66%±0.23^**^

Compared with control group, ^*^P<0.05, ^**^P<0.01.

### N25 induces autophagy in glioma cells and xenograft tumor model

**Figure 2:**
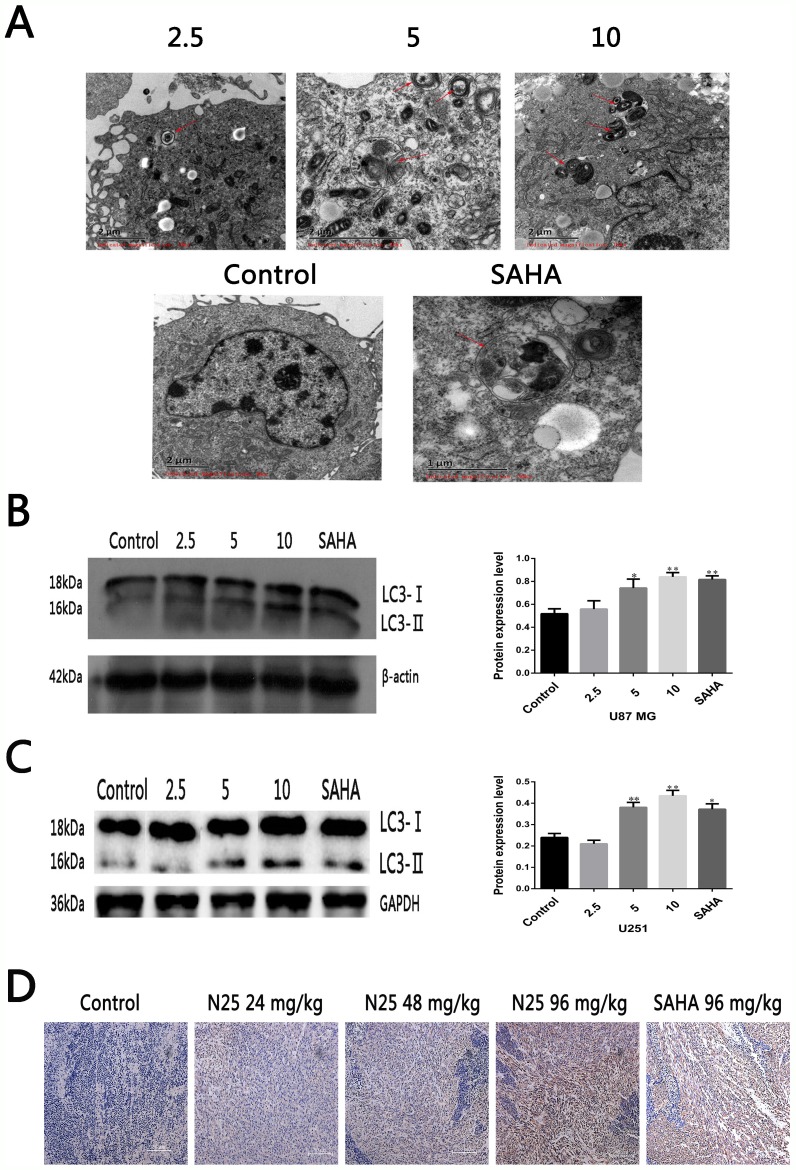
N25 induced autophagy in glioma cells and xenograft tumor model **(A)** N25 induced autophagosome formation in U87-MG cells treated with various concentration (2.5, 5, 10 μM) of N25 or 10 μM SAHA for 48 hours. The red arrows present autophagosome. **(B)** N25 up-regulated protein expression of LC3-II in U87-MG cells. **(C)** N25 up-regulated protein expression of LC3-II in U251 cells. **(D)**. N25 promoted LC3 protein expression in U87-MG xenograft tumor model. *P<0.05 and **P<0.01 compared with control group. The data are shown as the mean±SD (n=3), and representative results from three independent experiments with similar results are shown.

Several studies have suggested that SAHA can induce autophagy in certain human tumors [21, 23, 28–30]. We hypothesized that N25 can induce autophagy in glioma cells, such as U87-MG. Transmission electron microscopy (TEM) is a standard method to reliably detect autophagy. After U87-MG was treated with N25 for 48 hours, TEM was used to assess morphological changes in N25 treated cells (Figure 2A). Numerous autophagosomes were present in N25-treated cells, whereas the formation of autophagosomes was not seen in control group. In order to further conform the hypothesis that N25 can cause autophagy in glioma cells, we performed western blot analysis. After treatment with N25, there was an accumulation of LC3-II in U87-MG cells. Furthermore, we also demonstrated N25 can increase the expression of LC3 in xenograft tumor model by using immunohistochemistry (Figure 2D), which suggest that N25 can induce autophagy *in vivo*. Overall, our results showed that N25 can induce autophagy in both glioma cells and xenograft tumor model.

### The expression of HDAC3, Tip60 and LC3 in glioma samples

**Figure 3:**
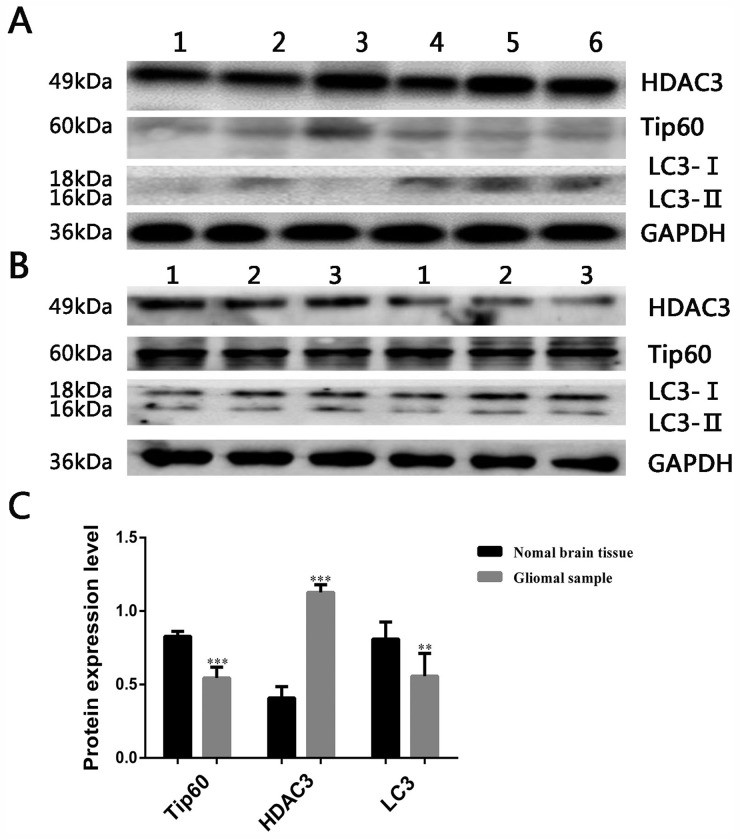
Expression of autophagy related protein in glioma samples **(A)** Expression of HDAC3, Tip60 and LC3 in glioma samples, the blots have been cropped and retain 6 bands. **(B)** Expression of HDAC3, Tip60 and LC3 in 3 normal brain tissues. **(C)** Protein expression bar graph in glioma samples and normal brain tissues. **P<0.01 and ***P<0.001 compared with normal brain tissue.

The expression of HDAC3, Tip60 and LC3 were measured in 20 glioma samples and 3 normal brain tissues. Western blot results revealed that the expression of HDAC3 was significantly higher in glioma samples than normal brain tissue, however, the expression of LC3 and Tip60 were lower compared with normal brain tissue (Figure 3A-3C).

### N25 inhibits HDAC3 and up-regulates Tip60

**Figure 4:**
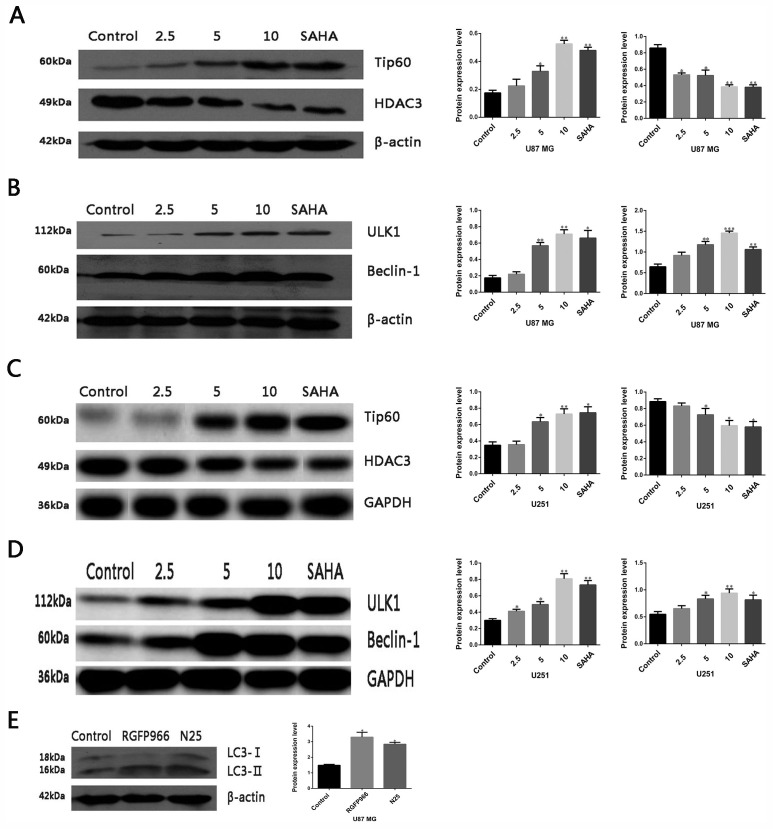
Protein expression after treatment with N25 in glioma cells **(A)** Expression of HDAC3 and Tip60 after treatment with various concentration (2.5, 5, 10 μM) of N25 or 10 μM SAHA for 24 hours in U87-MG cells. **(B)** Expression of ULK1 and Beclin-1 after treatment with various concentration (2.5, 5, 10 μM) of N25 or 10 μM SAHA for 24 hours in U87-MG cells. **(C)** Expression of HDAC3 and Tip60 after treatment with various concentration (2.5, 5, 10 μM) of N25 or 10 μM SAHA for 24 hours in U251 cells. **(D)** Expression of ULK1 and Beclin-1 after treatment with various concentration (2.5, 5, 10 μM) of N25 or 10 μM SAHA for 24 hours in U251 cells. **(E)** Expression of LC3 after treatment with selective HDAC3 inhibitor RGFP966 (10 μM) or N25 (10 μM) for 24 hours in U87-MG cells. *P<0.05, **P<0.01 and ***P<0.001 compared with control group. The data are shown as the mean±SD (n=3), and representative results from three independent experiments with similar results are shown.

To investigate the expression of HDAC3 and Tip60 after treatment with N25 in glioma cells, western blot analysis was carried out. Results revealed that N25 significantly inhibits the expression of HDAC3 and up-regulates the expression of Tip60 after treatment with N25 in U87-MG and U251 cells (Figure 4A, 4C).

### The expression of autophagy-related protein after N25 treatment

Activated acetyltransferase Tip60 is known to directly acetylates and stimulates the protein kinase ULK1 [25]. ULK1 induces autophagy by phosphorylating Beclin-1and activating VPS34 lipid kinase [26]. To clarify whether N25 also activates ULK1 and Beclin-1 after Tip60 up-regulated by N25, we performed western blot analysis. The western blot results revealed that expression of ULK1 and Beclin-1 was significantly higher after treatment with N25 in U87-MG and U251 cells (Figure 4B, 4D).

### Selective HDAC3 inhibitor RGFP966 induces autophagy in glioma U87-MG cells

To clarify N25 induces autophagy mainly associated with the inhibition of HDAC3, we applied a selective HDAC3 inhibitor RGFP966. Results showed that RGFP966 can increase the expression of LC3 in U87-MG cells (Figure 4E).

## DISCUSSION

Glioma is the most common primary brain tumor which has the highest mortality rate. Most patients die within 2 years of diagnosis. Novel therapeutic drugs and new treatment strategies are urgently needed.

Overexpression of HDACs in tumor cells is associated with cell proliferation [31–35]. Knockdown of HDACs can induce a range of antitumor effects such as cell cycle arrest, inhibition of proliferation, induction of apoptosis, differentiation and senescence, and disruption of angiogenesis [36]. These data suggest that HDACs may be an effective target for anti-cancer drugs.

A recent study implicated that HDAC3 in the pathogenesis of pediatric glioma due to its promotion of glioma cell proliferation and migration, additionally, the level of HDAC3 expression was associated with tumor grades [37]. In order to identify whether HDAC3 is over-expressed in adult gliomas, we collected 20 patient samples and measured HDAC3 protein expression by western blot. Compared with normal brain tissues, glioma samples contained elevated expressions of the histone deacetylate HDAC3 but decreased levels of the histone acetyltransferase Tip60.

In order to explore the influence of N25 on HDAC3 and Tip60 expression in glioma cells, U87-MG and U251 cells were treated with N25. We determined that N25 inhibits HDAC3 expression and up-regulates Tip60.

To explore the *in vitro* anti-tumor activity of N25 in glioma cells, we conducted a cell proliferation assay. The results demonstrated that the anti-tumor activity of N25 in U87-MG and U251 cells are stronger than SAHA. We additionally established a U87-MG xenograft tumor model and confirmed that N25 equally exhibited significant anti-tumor activity *in vivo*.

In order to further study the role of autophagy in glioma cells. We determined that LC3 is expressed at much lower levels in glioma samples than in normal brain tissues. This suggests that low level of autophagy is beneficial to glioma growth. Next, to investigate whether N25 induces glioma cells autophagy, we used TEM, a standard method to reliably detect autophagy, found that numerous autophagosomes were present in N25-treated cells. Further western blot experiment demonstrated that N25 (5 μM) significantly increased endogenous LC3-II accumulation and induced autophagy.

To examine whether N25 also activates ULK1 and Beclin-1 after up-regulated of Tip60 by N25, we performed western blot analysis. The western blot results revealed that expression of ULK1 and Beclin-1 was significantly higher after N25 treatment in U87-MG and U251. At last, to clarify N25 induces autophagy mainly associated with the inhibition of HDAC3, we applied a selective HDAC3 inhibitor RGFP966. The results showed that RGFP966 can increase significantly the expression of LC3 compared with control group in glioma U87-MG cells.

The study demonstrated that the anti-tumor activity of N25 in glioma cells is stronger than SAHA both *in vitro* and *in vivo* and may be mediated through induction of autophagy. Furthermore, HDAC3 was found to be significantly elevated and LC3 and Tip60 significantly decreased in glioma samples compared with normal brain tissues. Nevertheless, N25 inhibited HDAC3 and up-regulated the protein expression of Tip60, ULK1, and Beclin-1 in glioma cells. Meanwhile, after treatment with RGFP966 in glioma U87-MG cells, we found that autophagy induced by specific inhibition of HDAC3.

In conclusion, N25 has obvious anti-tumor activity *in vivo* and *in vitro* and through inhibition of HDAC3. N25 has slightly stronger activity than SAHA. N25 induces autophagy in glioma cells through a new mechanism of upregulating Tip60 by inhibiting HDAC3, causing upregulation of ULK1 (Atg1) and Beclin-1 (Atg6), and resulting in induction of glioma cell autophagy.

Through the research, we proposed and preliminarily demonstrated the possible mechanism of N25 induced autophagy in glioma cells on the basis of relevant reference. It was first proposed that HDACi induces glioma autophagy by inhibiting HDAC3. Meanwhile, this was the first time that we research the anti-tumor activity of HDACi N25 *in vivo* and the molecular mechanism of N25 induces glioma autophagy, and provided a possible direction for the treatment of glioma.

In this study, the results suggest that overexpression of HDAC3 in glioma cells is closely related to the level of autophagy. Other studies have confirmed that overexpression of HDACs is an important factor in tumorigenesis [10–12]. It also reported that HDAC3 overexpression is indeed exist in glioma and the proliferation of glioma cells was suppressed after knock-down of HDAC3 by siRNA [37]. Therefore, it is possible that overexpression of HDAC3 in glioma cells influences autophagy decrease and lead to glioma formation. As long as we changed the expression of HDAC3 in tumor cells and increase autophagy in glioma cells, it can lead to autophagic death of glioma cells. If glioma is this case, it can be used as a reference for glioma therapy that increasing autophagic cell death by inducing autophagy.

## MATERIALS AND METHODS

### Cell culture

Glioma cell line U87-MG and U251 were obtained from Sun Yat-Sen University Cancer Center. Cells were cultured in DMEM medium (Gibco, USA), supplemented with 10% fetal bovine serum at 37°C with 5% CO_2_.

### Proliferation assay

Cell Counting Kit-8 (yeasen, 40203ES80, China) was employed to determine the number of viable cells. In brief, 3×10^3^ cells/well were seeded into 96-well plates and allowed to adhere. After treatment with various concentrations of N25 or SAHA for 48 hours, 10 μL of CCK-8 solution was added to each well and the plates were incubated for another 2 hours. The absorbance was measured at 450 nm by Model 680 Microplate Reader (Bio-Rad, USA). The number of viable cells was counted by the absorbance. The value of IC50 was calculated by SPSS 22.0.

### Reagents

Vorinostat (SAHA, MK0683) was purchased from Dalian Meilun biotechnology. N25 were synthesized and identified according to the method in the literature [17]. RGFP966 (ApexBio, A8803, USA) was purchased from ApexBio.

### Antibodies

Anti-LC3B antibody 1:3000 (Abcam, ab51520), Anti-HDAC3 antibody 1:4000 (Abcam, ab32369), Tip60 Antibody 1:1000 (Cell Signaling, #12058), Beclin-1 Antibody 1:1000 (Cell Signaling, #3738), and Anti-ULK1 antibody 1:5000 (Abcam, ab128859) were utilized for experiments.

### Transmission electron microscopy

U87-MG cells were incubated with or without 100 mg/mL of PAMAM dendrimers G5 for 18 hours. Cells collected by trypsinization were fixed with 2.5% glutaraldehyde in 0.1 M PBS (pH 7.4) for 2 hours at 4°C and washed three times with 0.1 M cacodylate buffer containing 0.1% CaCl_2_. Samples were post-fixed with 1% buffered osmium tetroxide (pH 7.4) for 30 minutes. After dehydration using graded ethanol, the samples were polymerized at 60°C for 48 hours, and cut into ultrathin sections. They were then stained with uranyl acetate and lead citrate, and finally examined using a JEM 1230 transmission electron microscope (JEOL USA, Inc.).

### Western blot analysis

The human glioma samples were obtained from Sun Yat-Sen University Cancer Center, and the relevant analysis of glioma samples were approved by the Ethics Committee of Sun Yat-Sen University Cancer Center. Glioma tissue or cells were lysed using RIPA lysis buffer (Beyotime, China) containing 1% phenylmethanesulfonyl fluoride (PMSF) on ice for 30 minutes and then centrifuged at 12,000 RPM at 4°C for 15 minutes. The supernatants were collected and total protein concentrations determined by using BCA protein assay kit (Pierce, Rockford, IL, USA). We chose 8%, 10% or 12% SDS-gels according to protein molecular weight. After separation by SDS-PAGE, the proteins were transferred to polyvinylidene difluoride (PVDF) membranes (Millipore, USA). The membranes were blocked in western blocking buffer (Beyotime, China) for 2 hours, rinsed with western washing liquid (Beyotime, China) for 1 minute and incubated with the pertinent antibody at 4°C overnight. After washing with western washing liquid 3 times for 10 minutes, the membranes were incubated with horseradish peroxidase-conjugated secondary antibodies for 1 hour at room temperature. After development and fixing, the protein bands were analyzed by using Image J software, with β-actin or GAPDH as a control.

### Xenograft models

Animal handling procedures were approved by the Institutional Animal Care and Use Committee of Guangdong pharmaceutical university. BALB/c-nu mice 4-5 weeks of age and were purchased from the experimental Animal Center of Sun Yat-Sen University. They were maintained under specific pathogen-free conditions. Subcutaneous xenografts were established by injection of 3-5×10^6^ U87-MG cells per mouse to axilla (n=6 mice /group). Two weeks after inoculation, mice bearing similar size of tumors were randomly divided into five groups and then treated with one of three doses of N25 (24, 48, or 96 mg/kg), SAHA (96mg/kg) or control (0.9% NaCl). Tumor diameter was measured by vernier caliper and tumor volume was calculated according to relevant formula. After intragastric administration of drug for two weeks, the nude mice were sacrificed and the xenograft tumors from all groups harvested, dissected, and weighed.

### Immunohistochemistry

Specimens taken from the U87-MG xenografts were used for immunohistochemical detection of LC3. Sections of 4 μm in thickness were deparaffinized and rehydrated with xylene and graded alcohol solutions. After washing with PBS, endogenous peroxidase activity was quenched by 3% hydrogen peroxide, and the sections were boiled in 10 mM citrate buffer (pH 6.0) for 3–5 minutes in an autoclave sterilizer followed by cooling at room temperature for more than 30 minutes. After rinsing with PBS, the sections were incubated with primary antibodies overnight at 4°C. Sections were stained with related antibodies, respectively. After rinsing with PBS, the sections were incubated with PV6001 or PV6002 for 1 h at 37°C and stained with DAB for 2 minutes. The slides were counterstained with hematoxylin, dehydrated with graded ethanol, cleared with xylene, and mounted in neutral gum. All slides were analyzed by two independent observers.

### Statistical analysis

All values were presented as mean ± SD. Statistical analysis is carried out by one-way ANOVA using the SPSS statistical software (SPSS 22.0). Probability values (P-value) < 0.05 is considered as statistically significant.
